# Emerging Role of Tumor-Educated Platelets as a New Liquid Biopsy Tool for Colorectal Cancer

**DOI:** 10.34172/aim.2023.68

**Published:** 2023-08-01

**Authors:** Hossein Razzaghi, Milad Khabbazpour, Zohreh Heidary, Mohammad Heiat, Zeinab Shirzad Moghaddam, Parisa Derogar, Ahmad Khoncheh, Majid Zaki-Dizaji

**Affiliations:** ^1^Department of Laboratory Sciences, Faculty of Paramedicine, AJA University of Medical Sciences, Tehran, Iran; ^2^Human Genetics Research Center, Baqiyatallah University of Medical Sciences, Tehran, Iran; ^3^Vali-e-Asr Reproductive Health Research Center, Family Health Research Institute, Tehran University of Medical Sciences, Tehran, Iran; ^4^Baqiyatallah Research Center for Gastroenterology and Liver Diseases (BRCGL), Clinical Sciences Institute, Baqiyatallah University of Medical Sciences, Tehran, Iran; ^5^Endocrinology and Metabolism Research Institute, Tehran University of Medical Sciences, Tehran, Iran

**Keywords:** Colorectal cancer, Tumor-educated platelets, Diagnosis, Liquid biopsy, Biomarker

## Abstract

Colorectal cancer (CRC) is a major cause of cancer-associated death universally. Currently, the diagnosis, prognosis, and treatment monitoring of CRC mostly depends on endoscopy integrated with tissue biopsy. Recently, liquid biopsy has gained more and more attention in the area of molecular detection and monitoring of tumors due to ease of sampling, and its safe, non-invasive, and dynamic nature. Platelets, despite their role in hemostasis and thrombosis, are known to have an active, bifacial relationship with cancers. Platelets are the second most common type of cell in the blood and are one of the wealthy liquid biopsy biosources. These cells have the potential to absorb nucleic acids and proteins and modify their transcriptome with regard to external signals, which are termed tumor-educated platelets (TEPs). Liquid biopsies depend on TEPs’ biomarkers which can be used to screen and also detect cancer in terms of prognosis, personalized treatment, monitoring, and prediction of recurrence. The value of TEPs as an origin of tumor biomarkers is relatively new, but platelets are commonly isolated using formidable and rapid techniques in clinical practice. Numerous preclinical researches have emphasized the potential of platelets as a new liquid biopsy biosource for detecting several types of tumors. This review discusses the potential use of platelets as a liquid biopsy for CRC.

## Introduction

 Colorectal cancer (CRC) is the second deadliest cancer and the third most common cancer globally.^1,2^ During 2018, there were 1.8 million new patients of CRC and 881 000 deaths worldwide, which accounted for close to 10% of all new cancer cases and deaths.^1^ Unfortunately, 20% of newly diagnosed CRC cases are metastatic, and 25% of those with the localized disease will later develop metastases.^3^ The incidence and mortality rates are rising annually, and the growth of the population and the aging of society may further increase this incidence, posing a grave threat to public health.

 Up to now, the screening and detection of CRC generally depend on the assessment of serum biomarkers, tissue biopsy, and imaging.^4^ Nevertheless, recent studies demonstrated that the accuracy and sensitivity of imaging and pathological approaches are still restricted, and the specificity of the serum biomarkers is low. Also, as a major problem, it is challenging to perform repeated biopsies for early cancer detection, monitoring of disease progression, and further observation of tumor resistance mutations.^5^ Furthermore, a one-only biopsy typically does not represent a patient’s tumoral heterogeneity.^1^ Given that the survival of patients with CRC is extremely dependent on early detection and treatment, suitable biomarkers that can detect CRC and predict the therapy response in a timely fashion are urgently required.^6^

 The primary tumor tissue does not every time deliver sufficient evidence to distinguish individual patients for the purpose of maximally efficient treatment; so, it is imperative to develop a minimally invasive technique to screen high-risk people and identify the presence of CRC in asymptomatic cases at an early and treatable stage.^1,7^

## Liquid Biopsy

 Lack of early diagnostic methods and drug resistance are the most critical factors for the high mortality rate of cancer by indirectly inducing distant metastasis.^8^ With the fast advancement of isolation and gene identification technologies, the central role of liquid biopsy in tumor precision medicine has become ever more apparent.^9^ Liquid biopsies are samples involving blood, stool, urine, pleural fluid, saliva, and cerebrospinal fluid (CSF) collected for various analyses.^9,10^ Compared to tissue biopsy, liquid biopsy has advantages including easy accessibility and may cover the Mutational heterogeneity between cancer cells, whereas tissue biopsy is restricted to the changes in the tumor samples. Components of blood could supply a comprehensive real-time portrait of the tumor-connected alterations, which could be used for cancer screening, early diagnosis, and surveillance of dynamic variations in the molecular level of the tumor and resistance mechanism, which could powerfully aid oncologists in guidance of treatment and monitoring of patient’s response to treatment.^11^ Liquid biopsy as a new technique can be used to detect evidence of tumor secreted from a primary tumor and metastasis sites by investigating tumor-related molecular markers through circulating tumor cells (CTCs), circulating free (cf) DNA or RNA, circulating tumor DNA (ctDNA), tumor-educated platelets (TEPs), extracellular vesicles (EVs) such as exosomes, and circulating tumor-derived endothelial cells (CTECs).^9,12-14^ Currently, CTCs, ctDNA, exosomes, and TEPs have attracted most of the attention in the liquid biopsy research (Table 1).

**Table 1:** Characteristics of Liquid Biopsies

**Liquid Biopsy Component**	**Advantage**	**Limitation**	**Half-life**
CTCs	tumor cells allow for morphologic and molecular characterization of tumor, CTCs are live cells that can be used for drug screens and other functional assays	Scarcity (1-100 CTC per mL of blood), short half-life, physical and phenotypic diversity of the marker, difficulty in isolation, undetectable in many patients at early stage	1–2.5 h
ctDNA	Well-validated, reproducible technologies, mutation and epigenetic analysis of tumors	Only information from apoptotic or dead tumor cells, short half-life, undetectable in many patients at early stage	16 min
EVs	Exist in almost all body fluids, high concentration (~10^9^ particles/mL) in biofluids, high stability, secretion from living parental cells	Lack of high-quality isolation and component analysis devices, limited sensitivity, specificity, and low purity of current isolation methods	Several minutes to hours
TEPs	Easy access and isolation, abundance of RNA, long half-life	Lack of sufficient evidence	7-10 days

CTC, circulating tumor cell; ctDNA, circulating tumor DNA; EV, extracellular vesicle; TEP, tumor-educated platelet.

 CTCs are cells secreted from a primary and metastatic tumor into the circulatory system that can prepare valuable tumor-related information at the molecular level, including the DNA, RNA and protein. CTC testing in cancer patients as a real-time “liquid biopsy” can aid in tumor discovery, treatment monitoring, prognosis and personalized treatment.^15^ CTCs could independently predict survival in CRC, after adjusting for clinically important factors including the stage of cancer.^7,16^ CTCs are very rare because their half-life in the bloodstream is only 1.2 to 2.4 hours, and the majority of these cells are destroyed in the circulation because of physical and environmental damage such as shear stress, immune system attacks, and oxidative stress.^16-18^ Although CTC research may enable thorough investigations at the molecular level (RNA, DNA, and protein levels), their variability and paucity cause challenges in gaining a thorough knowledge of these cells.^19^ Generally, the small number of CTCs (~1-10) in the blood restricts their use and further technological improvement. However, whole genome sequencing and whole exome sequencing of CTCs ameliorate this restriction through unraveling the cancer heterogeneity.^9^ CTC-based cancer monitoring by the accounting of CTCs in the whole blood, CELLSEARCH^®^ System, was introduced to the clinic in cancer patients with metastatic breast, colorectal or prostate cancer. The presence of CTC detected by CELLSEARCH^®^ System is correlated with decreased overall survival and progression-free survival in patients.^20^ Building experimental models, *in vitro* and *in vivo*, using CTCs can provide a wealth of genetic and epigenetic data on the molecular biology of tumors and allow for the evaluation of sensitivity to anticancer medicines.^21^

 Circulating cell-free DNA (ccfDNA) are single and double-stranded DNA fragments ( ≤ 200 pb) secreted into the body fluids by apoptotic/necrotic cells. The ccfDNA secreted by cancer cells is called ctDNA and consists of a generally small fraction of the total ccfDNA in the blood.^22^ Since ctDNA cannot yet be differentiated from other circulating DNAs, its presence can only be determined by looking for tumor-specific mutations in cfDNA.^23^ Following blood collection, DNA released from dying blood cells taints and dilutes ctDNA. The main sources of cfDNA in healthy persons include the hematological system, the gastrointestinal tract, and the skin, but mostly white blood cells.^24^ CRC patients show greater levels of cfDNA than healthy people.^25,26^ In general, cfDNA examination can be used to detect point mutations or structural changes, microsatellite alterations, copy-number variations, cfDNA length variations, and methylation profile.^27^ Both CTCs and ctDNA are now used in standard clinical practice. CTCs testing allows for morphologic and molecular characterization of the tumor, while ctDNA examination is currently restricted to mutation discovery and epigenetic analysis.^28^ In CRC, ctDNA is a valuable liquid biopsy because it conveys numerous genetic changes that have similar genetic alterations as their origin tissue. Now, several ctDNA-based methods have been added to CRC screening, diagnosis and prognosis methods.^15^

 Exosomes (40–150 nm in diameter), a subset of EVs, are a heterogeneous class of vesicles with membrane that are actively secreted through a variety of cells and stably circulate in body liquids. They may pass the blood–brain barrier (BBB) and are found in various physiological fluids, including the CSF, plasma, urine, milk, amniotic fluid, and saliva.^29^ Exosomes, as novel tools of cell-cell communication, encompass a various spectrum of biologically active molecules, including nucleic acids (DNA, mRNA, lncRNA, miRNA, etc.), proteins and lipids, which mirror the composition of their originating cells.^30^ Exosomes can be found in nearly all body fluids, have high stability owing to the lipid bilayer, and transfer biological contents from the living parental cells.^31^ The exosomes derived from cancers are related to tumor growth, metastatic niches development, and evasion from the immune system.^32^ The diagnostic and prognostic potentials of exosomal biomarkers have been studied in several cancers. However, these cancer-related exosomes are just a minor portion of all exosomes existing in body fluids; therefore, highly sensitive detection methods are needed to isolate exosomes of interest with high efficiency and purity, in nanoscale size and intrinsic heterogeneity of exosomes.^31^ Currently, Exosome Diagnostics has introduced a few exosome-based tools for some cancers, including ExoDx^TM^ Lung (ALK) for detecting EML4-ALK fusion transcripts in the plasma of patients with non-small-cell lung cancer, ExoDx Prostate IntelliScore (EPI) as a risk assessment test for detection of high-grade prostate cancer, and MedOncAlyzer 170 a targeted pan-cancer panel for tumor profiling which examines both exosomal RNA and ctDNA. These promising tools need to be verified in larger clinical samples and populations before incorporation into routine clinical cancer diagnosis and treatment.

 Recently, TEPs have found particular clinical use for early prognosis, recurrence monitoring, guiding treatment and assessing its efficacy.^9,33^ It has been found that there is a different mRNA expression pattern in platelets between cancer cases and healthy controls due to the tumor cells and platelets interaction. These can be used as a biomarker for early discovery of cancer or metastasis. Current clinical trials evaluate the use of platelets as liquid biopsies to guide treatment in CRC patients. They will finally clarify whether platelets as liquid biopsies have the potential to predict the prognosis of CRC cases successfully. Cancer has been shown to affect platelet count, volume, activation status, platelet-derived proteins, and RNA content, but the impact of these alterations, especially at the molecular level, has not been incorporated to routine cancer screening, diagnosis and monitoring.

## Cancer and Platelets

 Platelets are small (2–5 μm), specialized, hematopoietic cells without nucleus produced from megakaryocytes that circulate in the blood and are the second most prevalent cell type in blood. The mean survival time of platelets is 7-10 days after shedding from megakaryocytes. These cells, in addition to their two major functions, hemostasis and thrombosis, have crucial functions in various physiological processes involving angiogenesis, wound healing, inflammation, immune responses, neurodegenerative diseases, and cancers.^34^

 Platelets have an important role in linking tissue damage/dysfunction and the inflammatory response during repair of injury; nevertheless, uncontrolled platelet activation results in chronic inflammation and consequently many pathological conditions, including cancer.^33^ Blood-based platelet parameters are potential biomarkers for many diseases, both acute and chronic, because of their easy accessibility and low-cost procedures of assessment.^35^ More frequently assessed platelet indices (PI) include the total platelet count (TPC), mean platelet volume (MPV) and platelet distribution width (PDW).

 Strangely, for a long time, platelets have been ignored in blood biomarker studies; however, increasing evidence demonstrated that platelets have a critical role in the development and progression of diseases, especially cancer.^36,37^ Augmented levels of platelet count and thrombopoietic factors are often noticed in patients with cancer. However, it is unknown which one comes first, an upsurge in thrombopoietic factors induces a rise in platelet count and then tumor growth stimulation, or a tumor releases thrombopoietic factors, which results in a rise in platelet counts.^38^ In the presence of occult cancer, platelet generation can be severely augmented following numerous tumor-derived and systemic factors.^36,39^ High TPC has been documented in various cancers^40^; this high TPC is related to systemic inflammation,^41^ and increased potential risk of tumor progression in CRC.^42^ Thrombocytosis in adults older than 40 years could be an indicator of cancer^43^ and nearly 40% of cases with unknown thrombocytosis (i.e. absence of iron deficiency or inflammatory disease) harbor some form of occult cancer.^44^ The association between TPC and cancer is well-documented. However, the impact of TPC as a biomarker of early-stage cancer is still uncertain.

 Over the lifetime of healthy people, platelet volume is quite constant but it can differ in a spectrum of diseases. It is demonstrated that platelets have elevated mitochondria and meaningfully smaller microtubules in patients with ovarian cancers in comparison to healthy people.^45^ MPV is shown to be increased in pancreatic cancer patients^46^ and raised MPV is an indicator of poor prognosis in several types of cancer including gastric cancer, pancreatic cancer and myelofibrosis.^47-49^ MPV, despite being an indicator of platelet size and activity, can be used as an inflammatory marker.^50^ Up to now, the effect of MPV in patients with cancer is not entirely elucidated. Raised MPV is associated with worse overall survival in cases with CRC^51^ However, reduced MPV level in cases undergoing chemotherapy is correlated with worse overall survival.^52^ Other studies showed that CRC patients, compared to the adenomatous polyp group, have higher MPV levels.^53,54^ Qian et alreported that in non-metastatic CRC, the pre- and post-treatment ratio of MPV is a prognostic factor for overall survival.^55^ Despite the contradictory findings of the MPV levels in cancer cases and healthy individuals, it is agreed by the majority of researches that reduced MPV is a good prognostic biomarker for cancer cases.^56^ Also, the impact of MPV on cancer screening seems limited to later stages of the disease.^38^ However, due to the slight difference between patients and controls, MPV as a sole factor does not appear to be a valuable marker.

 Some studies suggested that platelet indices compared to each other or other blood variables yield better results. Studies have shown that preoperative MPV/TPC in the peripheral blood of CRC cases is low,^53,57^ High levels of pretreatment TPC to lymphocyte count ratio (PLR) is associated with poor overall survival in CRC patients,^58^ and decreased TPC/PLR shows good prognosis in patients with oligometastatic colorectal cancer.^59^ MPV/TPC was associated with tumor infiltration and regional lymph node metastasis in CRC.^53^ TPC/PLR was associated with depth of infiltration,^60^ and this infiltration was associated with poor prognosis in postsurgical CRC patients.^61^ Nowadays, some prediction models, like the ColonFlag model, have been introduced that use blood-based parameters (including PLC), age and sex combined with machine-learning techniques to differentiate high-risk from low-risk CRC patients.^62,63^ These models give predict CRC patients with AUC > 0.75.

## Cancer and Tumor Educated Platelets

 During tumor development, tumor cells with direct interaction or through several released mediators, activate (educate) platelets by changing the RNA expression profile and proteome of platelets, resulting in “TEPs”. Following signals from tumor cells, platelets modulate the splicing of their pre-mRNAs, leading to variations in their transcriptome and molecular profiles.^64^

 Additionally, in interaction with tumor cells, platelets during their life cycle pick up biomolecules released from these cells and constantly absorb and enrich circulating free proteins, vesicles, nucleic acids and particles.^33^ The surface of platelets is crucial for communication between platelets and other cells in the plasma due to their active biomolecules on the surface. This indicates that platelets can be “educated” by numerous cell types, for example erythrocytes, leukocytes, megakaryocytes, and tumor cells.^65^

**Figure 1:**
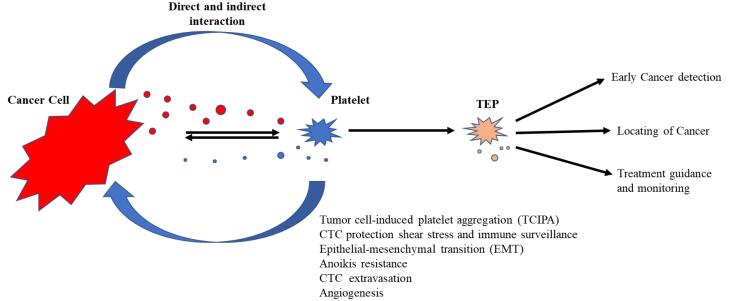
Activated Tumor-Educated Platelets (TEPs) Produced by Reciprocal Tumor-Platelet Interaction

 The role of TEPs in the progression and metastasis of numerous solid tumors is documented. Studies on nearly all different types of cancer have shown that the tumor induces activation of the clotting cascade through interactions with platelets.^9^ Cancer cells, through a key process called tumor cell-induced platelet aggregation, induce platelets to be activated, aggregated, and produce tumor favorite particles (Figure 1).^41^

 Cancer cells survive via platelet-mediated protection after entering the circulation system and subsequently, CTCs engage in extravasation, angiogenesis/vascular permeability and anoikis resistance.^34^ The communication between platelets and tumor is obviously mutual.^66^ Platelets were shown to have a stimulatory outcome on tumor progression,^36,37^ whereas tumor modulates multiple platelet features and functions and these alterations seem to be in the early stages of malignancy.^67-69^

 Platelets may communicate with other cells through discharging two types of EVs, exosomes (40–100 nm) and microparticles (100–1000 nm). Platelet-derived microparticles are the most prevalent EVs that exist in the blood.^70^ In physiological conditions, platelet-derived EVs are mostly generated from megakaryocytes^71^; however, induced platelet-derived EVs are associated with platelet activation.^72^ Medium-sized EVs (mEVs) secreted by thrombin-induced platelets of CRC cases showed different size distribution, and proteomic profiles compared to healthy subjects and stimulated prometastatic and prothrombotic phenotypes in tumor cells.^73^

## TEPs in Colorectal Cancer

 The interaction of platelets and cancers is complex and the mechanism has not been fully discovered. Plantureux et al demonstrated that the CRC tumor cells’ interaction with platelets generates chimeric EVs that inhibit early tumor growth through triggering macrophages, while promoting metastasis by epithelial-mesenchymal transition and endothelial activation.^74^ Recently, TEPs have been found to hold potential value as a promising liquid biopsy for several clinical and research uses.^75^ Platelet transcriptomes have numerous superiorities as a source for liquid biopsies in cancer management. Storing whole blood for up to 48 hours at room temperature does not have a significant impact on platelet RNA profiles. Abundant platelets are easily isolated and contain high-quality RNA. Distinct from nucleated cells with an active transcriptional machine, platelets transcriptomes are generally unchanged during the isolation process.^75^ Platelets are extremely reactive cells and may provide high sensitivity for longitudinal studies in cancer. TEPs can provide information on the existence, site, and molecular features of tumors by TEPs RNA splicing signatures.^76^ Although it is not completely discovered what factors exactly lead to differential transcriptional characteristics in platelets from patients with tumor, they are likely due to pick up of tumor-derived and circulating RNAs and reflect influences of the tumor microenvironment.^77,78^

 Most of the studies on the platelets of CRC patients have focused on mRNA expression analysis. These studies used RNA-seq to profile mRNAs of TEPs in CRC patients,^68,79,80^ and four studies used their data re-analysis and validation in CRC tissue,^81^ serum^82^ and platelets^83,84^ (Table 2). Best et al in 2015, in search for pan-cancer biomarkers, studied the potential of TEP mRNA profiles in several cancer patients and healthy donors from different cancer types. Using RNA-seq analysis, they could differentiate cancer patients from healthy volunteers. Additionally, this process precisely distinguished driver mutations, consisting of KRAS-, PIK3C4-, EGFR-, HER-2 and MET-positive tumors, proposing that TEPs may provide an accurate means for cancer detection and targeted treatments.^68^ Zou et al in 2022 constructed a platelet-based gene expression database, PltDB,^85^ that includes mRNAs, lncRNAs, miRNAs, and circRNA from 29 disease type (including CRC), 1,736 patients, and 672 controls from their previous RNA-seq data.^79^ They demonstrated that RNA profiles could be used for distinguishing and also cancer staging of CRC cases from other intestinal diseases unrelated to cancer.^79^

**Table 2:** Reported Studies in Tumor-Educated Platelets of CRC Patients

**Ref/Year**	**Samples**	**Method**	**Biomarker**	**Results**
2012 ^70^	Platelet & Plasma 35 CRC, 84 HC	ELISA	VEGF, bFGF, PDGF, PF4, TSP-1	VEGF, PDGF and PF4 high in platelet, not plasma
2015 ^71^	288 platelet samples from 6 cancer types including 41 CRC, 55 HC	RNA-seq	mRNA	Distinguishing cancer with 96% accuracy, identifying site of primary tumor with 71% accuracy GEO: GSE68086
2019 ^85^	Platelet 286 CRC (I66, II162, III23, IV27), 41 HC	Bioinformatic analysis (GSE68086) & qRT-PCR, functionally validation	mRNA	TIMP1 a potential independent diagnostic biomarker, platelets could carry RNA to CRC cells
2021 ^86^	Platelet 19 CRC, 4 HC	Bioinformatic analysis (GSE68086 and GSE89843), qRT-PCR	mRNA	7-gene prognostic model including RSL24D1 ↓, IFI27, HBD, CRYM, FCGR2A, IFITM3, and KLHDC8B ↑
2022 ^82^	1628 platelet samples from 18 cancer types including 85 CRC, 390 HC	RNA-seq	mRNA	Average 75% specificity, accurate detection in $\frac{2}{3}$ (stage I-IV), ½ (stage I-III), tumor site detection in 80% of patients GEO: GSE183635
2022 ^83^	Tissue Discovery (245 CRC), Validation (191 CRC)	Bioinformatic analysis (GSE161158, TCGA) of CRC-specific platelet-related genes	mRNA	10-gene prognostic model including TIAL1, C1orf198, PTPRN, CLK1, ANKRD13D, LSMEM1, ATP6AP1, SKAP1 ↑, ERAP1 and ANKRD17 ↓
2022 ^81^	Platelet, 132 CRC (I25, II48, III58, IV1), 190 HC	RNA-seq	mRNA	AUC = 0.92 BioProject Accession: PRJNA737596
2022 ^84^	Serum 105 CRC, 105 HC	Bioinformatic analysis (GSE68086) & qRT-PCR of Platelet-associated lncRNAs	LncRNA	LNCAROD, LINC00534, SNHG20, and TSPOAP-AS1 ↑ (both platelet and serum), AUC = 0.78

 Interestingly, one study investigated differentially expressed lncRNAs of CRC-platelets in the serum of CRC patients.^82^ They used RNA-seq data (GEO: GSE68086),^68^ to investigate differentially expressed lncRNAs in CRC-platelet samples. They then selected the four most overexpressed and the four most suppressed lncRNAs for final investigations. With a training set of 45 CRC (26 stage I/II, 18 stage III/IV) and 45 HC, and a validation set of 105 CRC (52 stage I/II, 52 stage III/IV) and 105 HC, they demonstrated that four lncRNAs, SNHG20, LNCAROD, LINC00534, and TSPOAP-AS1, were overexpressed in both the platelets and serum of CRC patients and these four lncRNAs can predict CRC with an AUC of 0.78; the expression levels of TSPOAP-AS1 and LNCAROD were associated with the staging of cancer and location of tumor.

 Platelets after activation can transfer many bioactive proteins into the microenvironment and uptake tumor-derived factors from the microenvironment; so the platelet proteome is significantly changed in cancer patients.^56^ Accordingly, PDGF, PF-4 and VEGF elevations were demonstrated in the platelets of CRC patients. These platelet-related proteins can be independent factors for predicting and significantly distinguishing CRC (AUC: 0.893, *P* < 0.001).

 In conclusion, in the therapy of CRC, early diagnosis, particularly the identification of precancerous adenomas, is believed to be crucial for improving patient survival. It is shown that common clinical features of platelets including TPC and MPV are changed in patients with cancer; however, their potential as diagnostic biomarkers for early cancer detection need extra investigations for confirmation. Liquid biopsy is a promising tool for cancer screening and early cancer detection and is an effective alternative or complement to the biopsy of tissue that enables the improvement of the overall survival of cancer patients. Generally, liquid biopsy is noninvasive, resolves heterogeneity of tumor and can provide real-time monitoring of cancer progression, treatment response or recurrence. Investigation of platelet (TEPs) has demonstrated that these cells could be a valuable tool for liquid biopsy of cancer patients, similar to other fluid biopsy tools (e.g. CTC, exosomes, ctDNA, cfRNA).

 The application of TEPs RNA profile in combination with artificial intelligence has shown high promise in the finding of CRC tumor and staging. However, the participation of TEP in CRC progression is not completely apparent, and their role as a tumoral marker requires further studies.

 Further protein and RNA profiles of TEPs from various cancers with different stages and long-term follow-up are needed for them to be used accurately with high sensitivity and specificity in the detection of early cancer and determining tumor stages. Also, combinatory analysis of TEP RNA with complementary sources, such as cfDNA/RNA and exosomes, will improve the detection of CRC in an early stage and help noninvasive disease observation. Imaging of platelet subcellular structures with super-resolution fluorescence microscopy is another advanced technology in the field of liquid biopsy that may open new horizons for clinical applications.^86^
